# Systemic treatment for hereditary cancers: a 2012 update

**DOI:** 10.1186/1897-4287-11-2

**Published:** 2013-04-01

**Authors:** Evgeny N Imyanitov, Tomasz Byrski

**Affiliations:** 1 Laboratory of Molecular Oncology, NN Petrov Institute of Oncology, St.-Petersburg, 197758, Russia; 2 Department of Medical Genetics, St-Petersburg Pediatric Medical University, St-Petersburg, 194100, Russia; 3 Department of Oncology, II Mechnikov North-Western Medical University, St.-Petersburg, 191015, Russia; 4 International Hereditary Cancer Center, Pomeranian Medical University, Szczecin, 70-111, Poland; 5 Clinic of Oncology, Pomeranian Medical University, Szczecin, 70-111, Poland

## Abstract

The history of specific therapy for hereditary tumors dates back to mid 1980s and involves a number of reports demonstrating regression of familial colon polyps upon administration of sulindac. Virtually no clinical studies on other hereditary cancer types were available until the year 2009, when Byrski et al. presented the data on unprecedented sensitivity of BRCA1-associated breast malignancies to cisplatin. This breakthrough has revived interest to the treatment of cancer in germ-line mutation carriers. Recent trials and clinical observations have confirmed the efficacy of platinating agents and PARP inhibitors in BRCA1/2-driven breast, ovarian and pancreatic carcinomas. Pegylated liposomal doxorubicin may be considered as a promising treatment option for BRCA1/2-related ovarian cancer after the failure of platinum-containing therapy. Several novel drugs have been recently introduced in the management of rare familial tumor syndromes. Vandetanib, a low-molecular weight RET kinase inhibitor, demonstrated substantial efficacy in the treatment of hereditary and sporadic medullary thyroid cancer. Vismodegib, an inhibitor of SMO oncoprotein, caused regression of basal-cell carcinomas in patients with Gorlin syndrome. Down-regulation of mTOR kinase by everolimus has been successfully used for the therapy of subependymal giant-cell astrocytomas in patients with tuberous sclerosis. The achievements in the prevention, diagnostics and treatment of hereditary cancers may serve as an excellent example of triumph of translational medicine.

## Introduction

1-5% of human cancers develop due to known germ-line defects. Virtually all major hereditary tumor types differ from their sporadic counterparts with respect to the underlying biological mechanisms, and thus may be considered as a somewhat distinct disease entity. First reports on specific therapy of familial tumors date back to mid 1980s [1]. It is getting increasingly apparent that cancers arising in mutation carriers often demonstrate peculiar spectrum of drug sensitivity [2]. Here we review recent advances and controversies in this field.

### Breast cancer

There are over 10 genes causing hereditary forms of breast cancer (BC), however only BRCA1- and BRCA2-related disease has been studied with sufficient level of comprehension. It is commonly stated that BRCA-driven malignancies are triggered by somatic inactivation of the remaining (wild-type) BRCA allele, thus providing an unique opportunity for a tumor-specific therapy. Indeed, while normal tissues of BRCA mutation carriers retain a non-altered copy of the gene, the transformed cells are characterized by complete loss of BRCA function. Absence of the BRCA1 or BRCA2 compromises DNA repair and increases sensitivity of the cell to particular DNA damaging agents [2,3].

Clinical studies on breast cancer demonstrated an unique sensitivity of BRCA1-accociated tumors to cisplatin [4]. The available literature describes 15 BRCA1 carriers treated by neoadjuvant cisplatin for BC, and 13 (87%) of them showed pathological complete response (pCR) [5-8]. First data on the use of cisplatin in metastatic setting have been published recently. Byrski et al. [9] observed objective responses in 16/20 (80%) patients, some of them heavily pretreated. Moiseyenko et al. [10] described a patient with BRCA1-related BC whose tumor did not respond to the first-line anthracyline-taxane therapy, but markedly regressed after administration of cisplatin. An experimental PARP1 inhibitor, olaparib, has also shown very encouraging results in both BRCA1- and BRCA2-driven BC, however its regulatory approval may take longer than initially expected [11].

Taxanes exert antitumor action via BRCA1-mediated apoptosis, therefore BRCA1-deficiency may mediate resistance to docetaxel or paclitaxel. Two systematic studies on BC provided strong support to this hypothesis. Kriege et al. [12] investigated taxane monotherapy for the treatment of metastatic BC disease, and described lower response rate and shorter progression-free survival in BRCA1-heterozygous patients as compared to BRCA2-related and sporadic cases. Byrski et al. [6] reported only 2/25 (8%) pathological complete responses in the BRCA1 patients treated by anthracycline-taxane (AT) combinations, while presumably less potent taxane-free anthracycline-containing regimens yielded 22% (11/51) pCRs. However, Arun et al. [13] recently presented the experience of neoadjuvant BC treatment in the MD Anderson Cancer Center, where BRCA1 carriers demonstrated high pCR rates for anthracycline-containing regimens both with and without taxanes (21/46 (46%) and 4/9 (44%), respectively). Entirely different outcomes of the AT therapy in the studies of Byrski et al. [6] and Arun et al. [13] deserve particular attention. It is essential to comment that while Byrski et al. [6] used the combination of doxorubicin and docetaxel for all described patients, run et al. [13] utilized a number of AT regimens; for example, some patients received distinct anthracycline (epirubicin) and/or taxane (paclitaxel) and/or were treated with the addition of 5-fluorouracil and/or cyclophosphamide.

A number of issues may be considered while designing the BC studies for the near future. The list of known BC genes is rapidly expanding, with the CHEK2 being apparently the most frequent cause of hereditary BC after BRCA1 and BRCA2. Drug response of CHEK2-related BCs has not been evaluated yet, neither in laboratory experiments nor in the patients [2]. Furthermore, the whole idea of selective chemosensitivity of BRCA-related BC is based on the “two-hit” hypothesis; however, some of the recent data indicate, that BCs arising in BRCA1 mutation carriers do not necessarily display the loss of the remaining allele, and haploinsufficiency of heterozygous BRCA1 cells may at least in some instances contribute to tumorigenesis [14-16]. It is of interest whether the actual somatic status of BRCA genes indeed influences the tumor response to the treatment. Most importantly, even highly BRCA-selective drugs, like cisplatin or PARP1 inhibitors, offer only a temporary tumor regression in the metastatic BC patients. It is hoped that intelligent combining of BRCA-specific compounds may offer significantly better outcomes [17]. The reported instances of cure of BRCA-mutated stage IV BCs by high-dose chemotherapy may deserve particular attention in this respect [18,19].

### Ovarian cancer

Approximately 15% of ovarian cancers (OCs) arise due to inherited BRCA1 or BRCA2 mutation. In addition, a significant portion of serous OCs demonstrate somatic inactivation of BRCA1/2 genes and therefore have similar biological properties [20]. BRCA deficiency explains sensitivity of OC to platinum-containing therapy and PARP1 inhibitors, which appears to be somewhat more pronounced in hereditary versus sporadic cases [21-23]. Prolonged drug treatment usually leads to the tumor resistance, which at least in some instances is attributed to the restoration of the BRCA gene function through the gain of second mutation [24].

The most noticeable achievement of the recent months is a convincing demonstration of high efficacy of pegylated liposomal doxorubicin (PLD) in BRCA-related ovarian cancer. PLD is an advanced formulation of doxorubicin, which is characterized by more favorable pharmacokinetic, pharmacodynamic and safety profiles as compared to conventional anthracyclines. PLD is included in the spectrum of drugs, which may be used for the treatment of ovarian cancer after failure of platinum-based therapy. Safra et al. [25] retrospectively compared the performance of 2^nd^- and 3^rd^-line PLD in proven BRCA1/2-carriers versus presumably non-hereditary OC patients; the objective response rates were 68% versus 49%, and the median time to treatment failure was 15.8 months versus 8.1 months. Adams et al. [26] considered PLD users with an average 2.7 prior chemotherapeutic regimens (range: 0-6), and also observed increased frequency of tumor responses (57% versus 20%) and prolonged progression-free survival (27.1 weeks versus 17.0 weeks) in BRCA mutation carriers. Kaye et al. [27] performed randomized comparison of PLD and olaparib in heavily pretreated BRCA-heterozygous OC patients; PLD arm demonstrated objective response rate of 18% and progression survival of 7.1 months, that was similar to the efficacy of the PARP1 inhibition. In contrast to PLD, topotecan, being also a standard therapeutic option for the previously treated ovarian cancer, showed null clinical benefit rate in BRCA mutation carriers [28].

A high-throughput pharmaceutical screen on BRCA2-deficient mouse mammary tumor cells pointed at potential efficacy of a well-known alkylating cytotoxic drug, melphalan [29]. In accordance with laboratory findings, Osher et al. [30] reported a patient with metastatic ovarian cancer, who received melphalan during 1 year in 1980s and remains disease-free for over 25 years.

### Pancreatic cancer

Pancreatic cancer (PC) is known for its resistance to virtually all available cytotoxic agents. However, 5% to 10% of PCs are caused by germ-line mutations in BRCA1, BRCA2 or PALB2 genes, and this subset of tumors may demonstrate significant sensitivity to DNA damaging agents and PARP1 inhibitors. There is a number of case observations supporting this assumption [31-33]. Two systematic studies on this issue have been reported in recent months. Lowery et al. [34] described 4 patients with advanced BRCA-related PC receiving PARP inhibitor alone or in combination with chemotherapy; 3 tumors responded to the treatment and 1 demonstrated stabilization of the disease. 6 patients from the same study underwent platinum-containing first-line therapy, and 5 of them showed response to the treatment. Faluyi et al. [35] described 11 BRCA-driven cases of metastatic PC; 2 complete and 3 partial responses were documented in 5 patients receiving platinum-containing drug combinations, while only 1 out of 6 PCs responded to gemcitabine.

### Colorectal cancer

Almost all colorectal cancers (CRCs), which arise in carriers of DNA mismatch repair (MMR) gene mutations, show instability of microsatellite repeats (MSI). MSI is also characteristic for a subset of sporadic CRCs. Both hereditary and sporadic MSI+ CRCs demonstrate reasonably favorable prognosis, probably due to immunogenicity of microsatellite unstable tumors. While familial CRCs are usually early-onset, sporadic MSI+ tumors are typical for elderly individuals. The latter category of MSI+ cancers is also characterized by the presence of BRAF mutations [36]. MSI test is technically easier that the detection of germ-line defects in MMR genes, therefore virtually all available studies consider MSI status without referring to inherited mutations in the MLH1, MSH2 or other candidates. Recently Sinicrope et al. [37] attempted to discriminate hereditary versus sporadic MSI+ CRCs based on patients age (younger versus older than 55 years) and, wherever possible, somatic BRAF status. This analysis led to suggestion that presumably hereditary stage III MSI+ CRCs do benefit from 5-fluorouracil-based therapy, while presumably sporadic ones do not.

There is an emerging class of targeted drugs, whose action is based on stimulation of local antitumor immune response [38,39]. They deserve to be considered for the future trials on MSI+ colorectal carcinomas, given a pronounced immunogenicity of this CRC subset.

### Medullary thyroid cancer

Hereditary forms of medullary thyroid cancer (MTC) develop due to inherited mutation in the RET oncogene and demonstrate pronounced sensitivity to the RET tyrosine kinase inhibitor, vandetanib [40]. Vandetanib studies were recently expanded to non-selected MTC, given frequent somatic RET alterations in sporadic tumors of this type [41]. The progression-free survival rate at 6 months was 83% for vandetanib versus 63% for placebo. Most of included patients had either confirmed RET mutation (hereditary or sporadic) or unknown RET status, and the former tended to fare better than the latter. This study led to the approval of the drug; interestingly, the European Medicines Agency recommends to evaluate RET mutation status while considering vandetanib therapy [http://www.ema.europa.eu/docs/en_GB/document_library/EPAR_-_Product_Information/human/002315/WC500123555.pdf], while the Food and Drug Administration states in the drug label that there is no evidence of a relationship between RET mutations and efficacy of this compound [http://www.accessdata.fda.gov/drugsatfda_docs/label/2011/022405s001lbl.pdf].

### Basal-cell nevus syndrome

Basal-cell nevus (Gorlin) syndrome is a rare hereditary disorder which is caused by mutation in the PTCH1 gene. PTCH1 inactivation abolishes its negative regulation of the SMO oncogene and thus initiates the growth of multiple basal-cell carcinomas. Clinical administration of a specific SMO inhibitor, vismodegib, prevented the appearance of new malignancies and resulted in the regression of existing neoplasms. None of the lesions progressed during the treatment, however the systemic tumor disease quickly relapsed after discontinuation of the therapy [42].

### Astrocytomas in tuberous sclerosis

Tuberous sclerosis is caused by germ-line mutations in TSC1 or TSC2 suppressor genes. Disruption of TSC1 or TSC2 leads to uncontrolled activation of mTOR kinase, and, consequently, to neoplastic growth. Tumors arising in patients with tuberous sclerosis are usually benign, however some of the neoplasms, especially if located in the brain, may cause severe disability and death. Recent development of specific mTOR inhibitors provided novel opportunities for the management of tuberous sclerosis. In particular, promising results have been achieved with everolimus: administration of this drug to the patients with serial subependymal giant-cell astrocytomas led to the marked tumor reduction in 21 (75%) of 28 enrolled patients [43].

### Conclusions and perspectives

Recent studies have convincingly demonstrated that hereditary and sporadic tumors may indeed require distinct treatment approaches. Ongoing revolution in technologies of DNA analysis, particularly the invention of next-generation sequencing, allows to expect that dozens of new familial cancer genes will be identified in the near future. Furthermore, dramatic increase of velocity and cost-efficiency of germ-line mutation testing provides the hope that virtually every cancer patient will soon be undergoing genetic examination right at the time of tumor diagnosis [44]. Advances in the management of hereditary cancer syndromes may serve as an excellent example of the power of translational medicine.

## Abbreviations

AT: Anthracycline-taxane combinations; BC: Breast cancer; CRC: Colorectal cancer; OC: Ovarian cancer; pCR: Pathological complete response; MMR: DNA mismatch repair; MSI: Microsatellite instability; MTC: Medullary thyroid cancer; PC: Pancreatic cancer; PLD: Pegylated liposomal doxorubicin

## Competing interests

The authors declare that they have no competing interests.

## Authors’ contributions

ENI and TB, drafted the manuscript. Both authors read and approved the final manuscript.
